# Design of a New Type of Compact Chemical Heater for Isothermal Nucleic Acid Amplification

**DOI:** 10.1371/journal.pone.0139449

**Published:** 2015-10-02

**Authors:** Kamal G. Shah, Dylan Guelig, Steven Diesburg, Joshua Buser, Robert Burton, Paul LaBarre, Rebecca Richards-Kortum, Bernhard Weigl

**Affiliations:** 1 Department of Bioengineering, Rice University, Houston, Texas, United States of America; 2 PATH, Seattle, Washington, United States of America; 3 Department of Bioengineering, University of Washington, Seattle, Washington, United States of America; Imperial College London, UNITED KINGDOM

## Abstract

Previous chemical heater designs for isothermal nucleic acid amplification have been based on solid-liquid phase transition, but using this approach, developers have identified design challenges en route to developing a low-cost, disposable device. Here, we demonstrate the feasibility of a new heater configuration suitable for isothermal amplification in which one reactant of an exothermic reaction is a liquid-gas phase-change material, thereby eliminating the need for a separate phase-change compartment. This design offers potentially enhanced performance and energy density compared to other chemical and electric heaters.

## Introduction

Nucleic acid based diagnostic tests enable earlier diagnosis for some infectious diseases compared to antibody tests [1]. Amplification of extremely low sample concentrations of RNA and DNA provide comparably high sensitivity and specificity [2]. Although PCR is typically used in laboratories in developed countries to amplify nucleic acids, the need for complex equipment, staff training, electricity, and precisely-timed thermal cycling limit its applicability to low-resource settings [3]. In response, isothermal amplification techniques such as loop-mediated amplification (LAMP), helicase-dependant amplification (HDA), cross priming amplification (CPA), and recombinase polymerase amplification (RPA) have been developed to amplify DNA to detectable concentrations in 15–60 minutes [4,5]. When combined with electricity-free isothermal chemical heaters that utilize an exothermic reaction with a phase-change material that melts at an appropriate temperature (55–65°C for LAMP and 15–37°C for RPA), these amplification techniques enable accurate diagnosis in low-resource settings where electricity is unreliable [4,6,7].

Given the large heat output of the reaction of magnesium metal with water to produce magnesium hydroxide and hydrogen gas as shown in Eq 1 [8], several devices have been developed based on this galvanic corrosion including ready-to-eat meals for soldiers and several isothermal chemical heaters, such as those developed by the PATH team and the Liu laboratory [9–11].

$${\text{Mg}}_{\left(\text{s}\right)}+2{\text{ H}}_{2}{\text{O}}_{\left(\text{l}\right)}\overset{{\Delta \text{H}}_{\text{r}}=-14.6\frac{\text{kJ}}{\text{g Mg}}}{\to }\text{Mg}{\left(\text{OH}\right)}_{2\left(\text{s}\right)}+{\text{H}}_{2\left(\text{g}\right)}$$ (1)

When the reaction temperature is moderated only by the boiling point of water, the resultant heat applied to the assay is too great; thus this reaction must also be coupled with a solid-liquid phase-change material (PCM) with an appropriate melting point, such as palmitic acid for LAMP (T_f_ = 61.9°C) to maintain an appropriate isothermal sample temperature [11, 12]. The additional presence of a PCM separate from the exothermic reaction increases device size, thus creating a design challenge to simultaneously minimize the ramp-up time to reach optimal LAMP temperatures, maximize the holdover time at optimal LAMP temperatures, and minimize device size. Up until now, electricity-free nucleic acid amplification temperature modulation has only been demonstrated with solid phase change materials separate from the exothermic reactants. In this work, we utilize the analogous exothermic magnesium reaction with methanol functioning as both reactant and PCM, thereby eliminating the need for having a separate PCM since methanol boils at 64.7°C (Eq 2) [13]:

$${\text{Mg}}_{\left(\text{s}\right)}+2{\text{CH}}_{3}{\text{OH}}_{\left(\text{l}\right)}\to \text{Mg}{\left({\text{CH}}_{3}\text{O}\right)}_{2\left(\text{s}\right)}+{\text{H}}_{2\left(\text{g}\right)}$$ (2)

The use of liquid-vapor phase change in lieu of the typical solid-liquid phase change used in isothermal chemical heaters may increase the ambient temperature operating range since the reaction temperature is capped by the boiling of the PCM. Additionally, enthalpy of vaporization is typically much higher than the enthalpy of fusion at a particular temperature, thus increasing energy density and allowing for a smaller device [9–13].

Due to the reduced thermal load of the system, the latter reaction may also enable a reduction in the ramp-up time and a potential increase in holdover time. Similarly, both reactions in Eqs 1 and 2 are accelerated in the presence of chloride ions [8]. Furthermore, since the holdover time is inversely related to the rate of reaction, combining the slow simmer reaction in Eq 2 with a kinetically fast reaction that brings the system to boiling may serve to reduce ramp-up time and potentially extend holdover time. Indeed, copper (II) chloride reacts exothermically at a rate 10 orders of magnitude faster than the chloride ion catalysed magnesium-water reaction in accordance with Eq 3 [7,8]:

$${\text{Mg}}_{\left(\text{s}\right)}+{\text{CuCl}}_{2\left(\text{aq}\right)}\overset{{\Delta \text{H}}_{\text{r}}=-24.0\frac{\text{kJ}}{\text{g Mg}}}{\to }{\text{Mg}}_{\left(\text{aq}\right)}^{2+}+2{\text{ Cl}}_{\left(\text{aq}\right)}^{-}+{\text{Cu}}_{\left(\text{s}\right)}$$ (3)

Therefore, we explored the impact of chloride ion and copper (II) chloride concentration on the ramp-up and holdover time of a proof-of-concept liquid-vapor PCM isothermal chemical heater that is compatible with LAMP.

## Materials and Methods

Design criteria encompassing desirable performance and form factor were established in accordance with the requirements of LAMP, such as for foot-and-mouth disease, and of laboratories in developing countries. Given the challenges of transporting materials in resource-limited settings, the reusable components of the proof-of-concept prototype were designed for fabrication solely using materials that would be locally available in a low resource setting and using common laboratory equipment with no reliance on additional custom molded or printed components. An average ramp-up time to 57°C of less than 5 minutes and a holdover time of 15 minutes for temperatures between 57 and 62°C was defined *a priori* to characterize a successful prototype.

**Fig 1:**
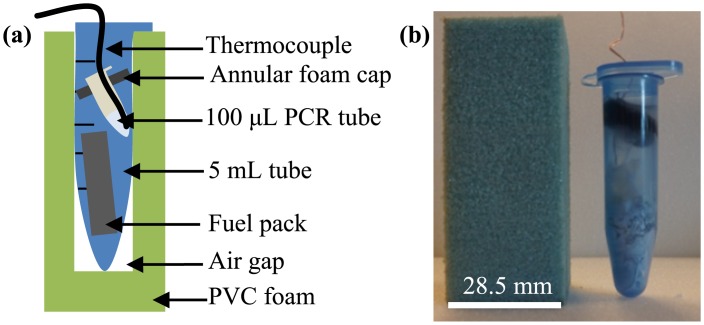
Device design. **(a)** Labeled vertical cross-section and **(b)** photograph of used, proof-of-concept, direct liquid-vapor phase change, and isothermal chemical heater. The exothermic reaction occurs in a standard laboratory 5 mL tube and is initiated by the addition of methanol to the tube.

In light of these design specifications, six identical proof-of-concept prototypes were constructed as shown in Fig 1 by drilling a 9/16” diameter, 60 mm hole in a 28.5 mm x 28.5 mm x 70 mm reusable PVC foam block (Item 85925K352, McMaster-Carr). A standard 5 mL centrifuge tube (Eppendorf North America Biotools) was pressed into the cavity and used as a disposable reaction chamber, and a pressure-equalizing hole was drilled into the cap with a 0.067” diameter drill bit. A 20 mm x 32 mm fuel pack was constructed by heat-sealing muslin (Item 10011, Muslinbag) that contained 400–450 μm particle diameter, 500 ± 2 mg, mechanically alloyed magnesium-iron (Luxfer Magtech Inc, USA) and 0, 10, 25, 50, 75 or 100 ± 2 mg sodium chloride (Item S7653, Sigma-Aldrich). In a second set of experiments, with 50 mg of sodium chloride mixed into the fuel pack, 0, 10, 20, 30, 40, 50 or 60 ± 2 mg anhydrous copper (II) chloride (Item 22011, Sigma-Aldrich) was added to the reaction chamber to prevent premature corrosion (50 mg of copper (II) chloride is needed to bring the system to boiling). An open-cell annular foam PCR tube holder was placed at an angle at a fixed height in the centrifuge tube to ensure that a 100 μL PCR tube was consistently positioned in all tests.

In all performed tests, the PCR tube contained 50 μL of tap water and was instrumented with a T-type thermocouple sampled continuously at 1 Hz with a custom LabVIEW virtual instrument via a NI cDAQ–9172 and NI–9211 (National Instruments). The isothermal heater was initiated by injecting a 3 g bolus of methanol to the reaction chamber at an ambient temperature of 23°C. To simulate use at the point-of-care, device performance was evaluated with 50 mg of sodium chloride in an environmental chamber (BTL–433, ESPEC, Hudsonville, Michigan) at an ambient temperature set to 22 or 45°C. All quantities are reported as a mean ± standard deviation ($\bar{\text{X}}$ ± s) unless otherwise indicated. Statistical analysis was performed by comparing the ramp-up and holdover times in each experimental group to a negative control via an unpaired, one-tailed t-test with unequal variance.

## Experimental Results

The six identical proof-of-concept prototypes successfully met the requirements for LAMP with holdover times of 19.0 ± 5.0 minutes when the fuel pack was mixed with 50 mg of sodium chloride. However, the ramp-up time was slightly longer than the 5 minutes targeted, and was 7.9 ± 1.9 minutes.

**Fig 2:**
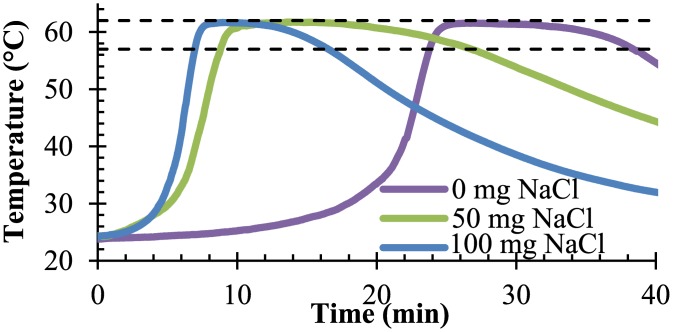
Representative temperature-time curves. The isothermal chemical heater shows relatively flat temperature-time profiles between 57°C and 62°C (dashed lines).

**Fig 3:**
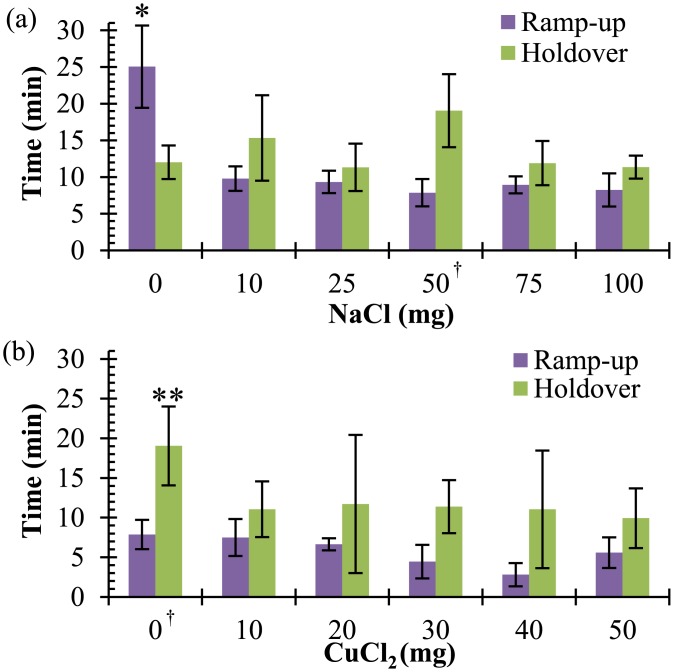
Ramp-up and holdover times. **(a)** Ramp-up times ($\bar{\text{X}}$ ± s) are reduced (*p = 0.018) and holdover times ($\bar{\text{X}}$ ± s) show no change with the addition of sodium chloride to the magnesium-iron fuel pack. (n = 3) **(b)** In contrast, the addition of copper (II) chloride does not significantly affect ramp-up, but significantly reduces holdover (**p = 0.005). (n = 3) **†** indicates identical data points.

Fig 2 shows characteristic temperature-time profiles for the chemical heaters at varying levels of sodium chloride. As expected and shown in Fig 3a, there is a statistically-significant decrease in ramp-up time from 25.0 ± 5.6 minutes to 8.9 ± 1.5 minutes when comparing the negative sodium chloride control to all tested sodium chloride concentrations (p = 0.018). No statistically significant difference in holdover time was observed with the addition of sodium chloride (p = 0.32), and was 12.7 ± 3.4 minutes. Temperatures exceeding the 62°C upper threshold by up to 0.1°C were observed when 75 or 100 mg of sodium chloride was added.

In contrast, the addition of copper (II) chloride resulted in no statistically-significant difference in ramp-up time compared to the negative control as shown in Fig 3b (p = 0.06). Upon addition of copper (II) chloride, holdover time decreased from 19.0 ± 5.0 minutes to 10.6 ± 4.7 minutes (p = 0.005). Overheating by up to 0.9°C was observed when 30 mg or greater of copper (II) chloride was added.

**Fig 4:**
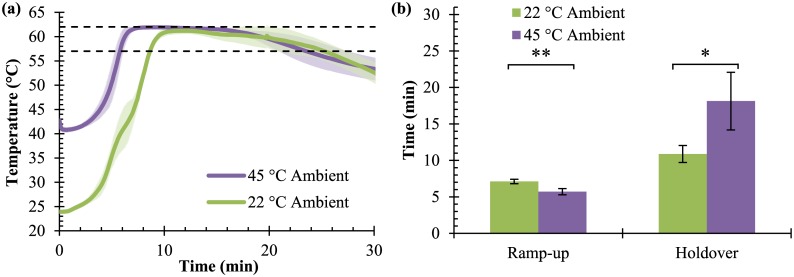
Performance at elevated temperatures. **(a)** The temperature-time curve ($\bar{\text{X}}$ ± s) shows smoother ramp-up and extended holdover between 57°C and 62°C (dashed lines) at a 45°C ambient temperature compared to 22°C. (n = 3) **(b)** Ramp-up time decreases by 1 minute (**p = 0.006) and holdover time increases by 60% compared to room temperature (*p = 0.04). (n = 3).

As shown in Fig 4, increasing the ambient temperature from 22 to 45°C led to a statistically significant, though small, decrease in ramp-up time from 7.1 ± 0.3 minutes to 5.7 ± 0.4 minutes (p = 0.006). The holdover time increased from 10.9 ± 1.2 minutes to 18.1 ± 4.0 minutes (p = 0.038).

The device is also compatible with non-methanol liquid-vapor PCM such as water, but ramp-up (to 90°C) and holdover time (between 90 and 95°C) is reduced to 29 seconds and 117 seconds, respectively (data not shown).

## Discussion

Isothermal nucleic acid amplification shows significant promise to enable early diagnosis compared to antibody-based tests in the developing world. However, current compact solid-liquid phase change material-based systems may be impractical to design to operate at large ambient temperature ranges. Although the Liu chemical heater is operable between 20 and 40°C, the single-use device is complex and only compatible with sample volumes up to 15 μL, which may be impractical for larger sample volumes such as when diagnosing HIV [10, 14]. A disposable, solid-liquid phase-change, isothermal heater developed by LaBarre et al. occupies a 85 cm^3^ volume but is only operable over a 14°C ambient temperature range [9].

In contrast, a liquid-vapor PCM also used as reactant would enable use at ambient temperatures of up to the boiling point, thereby increasing the feasibility of designing a device suitable for the developing world. The heater described in this work occupies a volume of 57 cm^3^, weighs about 15 g, requires only a drill to manufacture, and is compatible with samples up to 100 μL. The external foam housing is reusable and the device costs just $1.61 for the housing and $0.41 per use at low manufacturing volumes. In comparison, an alkaline D-cell battery alone occupies a volume of 56.5 cm^3^, weighs 135 g, and costs $0.55 (McMaster-Carr).

One potential concern for using liquid-vapor PCM is the altitude-dependence of the boiling point. For example, water boils at 92°C in Addis Ababa, Ethiopia (elevation of 2360 meters). However, this apparent limitation may instead enable precise control of the temperature profile if pressure-dependent valves are incorporated into future liquid-vapor PCM devices. By operating at a precisely controlled pressure, a liquid-vapor PCM device may improve holdover time by taking advantage of the increase in temperature when a gas is pressurized.

An additional benefit of liquid-vapor PCM heaters may be their adaptability to a variety of operating temperatures for further use for sample lysis, DNA denaturation, reagent reconstitution, organism culture or other diagnostic applications. In a solid-liquid PCM device, the entire device may have to be re-engineered to be compatible with a new PCM in order to supply a different target temperature. The fuel mass would also need to be adjusted to ensure that the latent heat absorption of the PCM is sufficient to absorb the heat emitted by the exothermic reaction. In contrast, since the liquid-vapor PCM reacts directly with the magnesium fuel in a two-to-one molar ratio, alcohols with higher boiling points, such as ethanol (T_b_ = 78°C), may be directly substituted in lieu of methanol with no change to device geometry or manufacturing. However, holdover time is reduced when water is substituted for methanol, which may limit the usefulness of direct alcohol substitution. Liquid-vapor systems may be somewhat limited by these appropriate material constraints but utilizing azeotropes or dilutions may offer a level of adjustability to the systems.

Since we did not observe substantial gains to system performance when adding more than 10 mg (0.28% by mass) of sodium chloride, there appears to be a saturation of reaction rate after a critical threshold of sodium chloride is reached. Given the toxicity of copper (II) chloride, the reduction in holdover time, and the tendency to overheat, copper (II) chloride may not be appropriate to include in the chemical heater for use in the developing world. We have therefore demonstrated the efficacy of a sodium chloride-catalyzed, isothermal, exothermic chemical heater based on the methanol-water reaction at temperatures compatible with LAMP and potentially with other isothermal methods such as HDA and CPA, which uses the Bst polymerase and are most efficient in the range 60–65°C [5].

Despite the challenges present in developing a liquid-vapor PCM, isothermal heater, there is significant potential for such a device to improve access to diagnostics in the developing world. Unlike other isothermal chemical heaters, the proposed device has great potential to simplify device design and manufacturability by eliminating the need for a separate PCM; alleviate ambient temperature range concerns with a liquid-vapor PCM; and increase the likelihood of successful uptake by lowering barriers to materials access and precise machining requirements [9].

## Conclusions

We have demonstrated that a low-cost, liquid-vapor PCM exothermic isothermal heater may be designed for use with isothermal amplification at temperatures required for LAMP, HDA, and CPA. Such a device may be suitable for use in developing countries by being simple and reduce device volume and mass compared to previous chemical or electric heaters, while expanding the ambient temperature operating range.
